# Redirecting differentiation of mammary progenitor cells by 3D bioprinted sweat gland microenvironment

**DOI:** 10.1186/s41038-019-0167-y

**Published:** 2019-09-23

**Authors:** Rui Wang, Yihui Wang, Bin Yao, Tian Hu, Zhao Li, Yufan Liu, Xiaoli Cui, Liuhanghang Cheng, Wei Song, Sha Huang, Xiaobing Fu

**Affiliations:** 1 Tianjin Medical University, Tianjin, 300070 People’s Republic of China; 2 Key Laboratory of Tissue Repair and Regeneration of PLA, and Beijing Key Research Laboratory of Skin Injury, Repair and Regeneration, Fourth Medical Center of General Hospital of PLA, Beijing, 100048 People’s Republic of China; 3 Wound Healing and Cell Biology Laboratory, Institute of Basic Medical Sciences, General Hospital of PLA, Beijing, 100853 People’s Republic of China

**Keywords:** 3D bioprinting, Artificial microenvironment, Differentiation, Mammary progenitor cells, Sweat gland, Extracellular matrix, MPC, ECM

## Abstract

**Background:**

Mammary progenitor cells (MPCs) maintain their reproductive potency through life, and their specific microenvironments exert a deterministic control over these cells. MPCs provides one kind of ideal tools for studying engineered microenvironmental influence because of its accessibility and continually undergoes postnatal developmental changes. The aim of our study is to explore the critical role of the engineered sweat gland (SG) microenvironment in reprogramming MPCs into functional SG cells.

**Methods:**

We have utilized a three-dimensional (3D) SG microenvironment composed of gelatin-alginate hydrogels and components from mouse SG extracellular matrix (SG-ECM) proteins to reroute the differentiation of MPCs to study the functions of this microenvironment. MPCs were encapsulated into the artificial SG microenvironment and were printed into a 3D cell-laden construct. The expression of specific markers at the protein and gene levels was detected after cultured 14 days.

**Results:**

Compared with the control group, immunofluorescence and gene expression assay demonstrated that MPCs encapsulated in the bioprinted 3D-SG microenvironment could significantly express the functional marker of mouse SG, sodium/potassium channel protein ATP1a1, and tend to express the specific marker of luminal epithelial cells, keratin-8. When the Shh pathway is inhibited, the expression of SG-associated proteins in MPCs under the same induction environment is significantly reduced.

**Conclusions:**

Our evidence proved the ability of differentiated mouse MPCs to regenerate SG cells by engineered SG microenvironment *in vitro* and Shh pathway was found to be correlated with the changes in the differentiation. These results provide insights into regeneration of damaged SG by MPCs and the role of the engineered microenvironment in reprogramming cell fate.

**Electronic supplementary material:**

The online version of this article (10.1186/s41038-019-0167-y) contains supplementary material, which is available to authorized users.

## Background

Mammary progenitor cells (MPCs) provide one kind of ideal tools for studying engineered microenvironmental influence due to its accessibility and continually undergoes postnatal developmental changes. It can gradually differentiate into many kinds of mammary gland cells after delivery and undergo many rounds of proliferation and apoptosis during the life [1]. In addition to the role of hormones, the local tissue microenvironment also plays a key role in the fate of MPCs [2, 3]. The maintenance and differentiation of MPCs can be achieved by perceiving signals from the components of extracellular matrix (ECM). For example, investigators have discovered that laminin I could maintain MPCs in a quiescent state and MPCs could differentiate into basal cells by the expression of the P-cadherin in the ECM [4].

There is increasing evidence that natural microenvironment has become one of the key factors affecting cell behavior and function in developmental, physiological, and pathological processes [5]. Some researchers have transplanted the mammary epithelium into the salivary gland mesenchyme, resulting in a structure similar to the salivary gland epithelium [6]. When co-cultured with mammary mesenchyme, the salivary epithelium could develop a mammary gland-like ductal tree which could even respond to hormonal stimuli [7]. Other research has transplanted sweat gland (SG) progenitor cells into the mammary glands of lactating mice and found that they expressed milk proteins [8]. These previous experiments demonstrated the dominance of the mammary niche over the phenotype of cells from other tissues; while investigations involving MPCs reprogramming to regenerate foreign tissues are seldom reported.

The artificial microenvironment, which imitates natural microenvironment *in vitro*, has recently emerged as a significant field in regenerative medicine [9]. It plays a vital role in tissue regeneration *in vitro*, especially those tissues or organs that cannot be completely regenerated after injury. A crucial aspect of the cell-laden artificial microenvironment is that the bioink must be cytocompatible, which restricts the choice of materials [10, 11]. Researchers have so far designed and manufactured many types of artificial microenvironments using multiple hydrogels [12–14]. However, the complexity of natural microenvironment cannot be completely replaced only by those materials [15]. Interactions between cells and ECM are so complicated that a tissue-specific microenvironment is necessary to sustain the cells regeneration *in vitro* [15]. Therefore, we use gelatin-alginate hydrogels which have good cell compatibility combined with the components from mouse SG-ECM proteins to fabricate a tailored bioink. At present, the mainstream three-dimensional (3D) bioprinting approach is used to build a 3D construct which can imitate the natural 3D microenvironment [15–18]. A large number of our previous studies prove that 3D bioprinted scaffolds benefit SG regeneration [19–21]. Here, we creatively produce an artificial SG microenvironment via combining the advantages of our tailored bioink and 3D bioprinting approach to research the regeneration of SG cells *in vitro*.

Although both the mammary glands and SG originate from epidermal progenitor cells, their functions are quite different. Mature mammary glands have the function of secreting milk to feed offspring while SG can perspire to maintain the homeostasis by regulating body temperature [22]. That led to the question of whether or not the 3D bioprinted SG microenvironment has the ability to redirect the differentiation of MPCs. Two-dimensional (2D) cell culture systems could not offer an ideal setup to study highly branched cells such as glandular cells. In 2D cultures, the growth of SG cells is unrealistically flattened, limiting the acquisition of full cellular functionality, and the cellular microenvironment is poorly modeled. In this work, we have cultured MPCs in the 3D bioprinted SG microenvironment and results of immunofluorescence and quantitative real-time PCR analysis have shown that induced MPCs express the functional protein marker of luminal epithelial cells of SG.

The development of SG is regulated by a relay of signals initiated by Wnt/β-catenin with subsequent participation of EDA/EDAR/NF-κb and Shh pathways. The induction of SG is controlled by Wnt/β-catenin and duct formation involves EDA/EDAR/NF-κb. Shh is downstream of Eda and regulates final secretory region formation [23, 24]. But Shh signaling pathway does not participate in the development of MPCs morphology and expression of function [25, 26]. In this study, the Shh pathway was detected and found to be correlated with the redirection of MPCs to SG cells.

## Methods

### Isolation and identification of MPCs

Pregnant day 12–14 (P12–14) C57BL/6 mice were purchased from SPF Biotechnology Co., Ltd. (Beijing, China). Isolate 2nd, 3rd, 4th, and 5th pairs of mammary glands and washed them with phosphate-buffered saline (PBS) (ZSGB-BIO, Beijing, China) for five times. They were cut into the consistency of sludge (about 10 min of continuous scissoring) and digested with 2 mg/ml collagenase I (Solarbio, Beijing, China) at 37 °C for 90 min with shaking every 5–10 min. Centrifuged at 1500 rpm for 5 min to collect sediments and washed them with DMEM/F12 (Gibco, USA) supplemented with 10% fetal calf serum for 3 times. The cells were cultured with conditional medium (DMEM/F12 with 5% fetal calf serum, 10 ng/ml epidermal growth factor, 1% ITS Liquid Media Supplement (Sigma, USA), 1% penicillin-streptomycin mixture, 0.4 μg/ml hydrocortisone, 2 ng/ml triiodothyronine). MPCs were identified by immunofluorescence co-location technique. After being cultured for 3 days, the cells were fixed in 4% paraformaldehyde (PFA) for at least 30 min. The cells were washed 2 times for 3 min with PBS and blocked for 30 min with 0.3% Triton X-100%. Then washed the cells with PBS once more. After permeabilization with 5% goat serum (Zsbio, China) at 37 °C for 30 min, the cells were incubated with primary antibodies overnight at 4 °C. After the cells were washed twice with PBS, they were incubated with second antibodies for 2 h at room temperature. Finally, the cells were incubated for 10 min with DAPI (1:300, Beyotime) as a nuclear stain. The antibodies used were as follows: keratin-14 (K14) (mouse, 1:200, Abcam), keratin-19 (K19) (rabbit, 1:200, Abcam), and goat anti-mouse Alexa Flour 488 (1:300, Beyotime), goat anti-rabbit Alexa Flour 594 (1:300, Beyotime). In order to screen differential expression markers between MPCs and SG cells, we isolated SG cells of C57BL/6 mice [27] and detected the expression of ATP1a1, ATP1b1, keratin-5 (K5), and K19 in both SG cells and MPCs by the above methods. The antibodies used were as follows: ATP1a1 (rabbit, 1:200, Abcam), ATP1b1 (rabbit, 1:200, Abcam), and K5 (mouse, 1:200, Abcam). All animal procedures were approved under the guidelines of the Institutional Animal Care and Use Committee of Chinese PLA General Hospital (Beijing, China) (approval number SCXK(BJ)2017-0001).

### The preparation of mouse SG-ECM proteins

Four feet of C57BL/6 mice aged 1 day were cut and ground into a paste. Attenuated them with PBS and subsequently centrifuged at 4 °C 13000 rpm for 5 min to collect the supernatant, called dermal homogenates. The mouse SG-ECM proteins were contained in dermal homogenates.

### Synthesis of *in vitro* 3D bioprinted SG microenvironment

The 3D bioprinted SG microenvironment was fabricated by a bioprinting platform (Regenovo 3D Bio-printer, China) based on rapid prototyping technology. It can print ideal complex 3D structures in designated places with live cells and biomaterials. The gelatin (Sigma, 96 kDa, type B) and sodium alginate (Sigma, 75–100 kDa, guluronic acid 39%) were dissolved into PBS in a ratio of 3:1 to form homogeneous composite hydrogels and then sterilized by pasteurization. A mixture of suspended cells and mouse SG-ECM proteins was added into the composite hydrogels in a concentration of 10% to fabricate the tailored bioink after the composite hydrogels cooled to 37 °C. There were 1.5 million cells per milliliter of composite hydrogels. Then put the bioink into a sterile syringe and printed as a cylinder with a grid inside.

The experiment consisted of four groups: non-protein (MPCs were added into the composite hydrogels which contained gelatin and sodium alginate without mouse SG-ECM proteins and then printed in a cylinder with a grid inside); non-bioprinted (MPCs and mouse SG-ECM proteins were added into the composite hydrogels which contained gelatin and sodium alginate without printed); SG-ECM (MPCs and mouse SG-ECM proteins were added into the composite hydrogels which contained gelatin and sodium alginate and then printed in a cylinder with a grid inside); SG-ECM+In (MPCs and mouse SG-ECM proteins were added into the composite hydrogels which contained gelatin and sodium alginate and then printed in a cylinder with a grid inside. The inhibitor of Shh signaling pathway (MCE, USA) was added into the conditional medium in a working concentration of 20 nM/ml).

Finally, each group was cross-linked with 2.5% CaCl_2_ for 10 min (to crosslink the alginate) at room temperature and washed with DMEM (Gibco, Canada), then cultured with conditional medium in a CO_2_ incubator at 37 °C. Observed every group by fluorescence microscopy (Leica BMI4000, Germany) after cultured 1, 3, 7 and 14 days.

### Physical properties and cell viability of the 3D bioprinted SG microenvironment

The 3D bioprinted SG microenvironment was photographed under scanning electron microscopy (SEM S-4800, HITACHI, Tokyo, Japan) to observe their pore structures after dehydration and measured the pore sizes. Cell viability of the 3D bioprinted SG microenvironment was observed by using LIVE/DEAD^®^ Viability/Cytotoxicity Kit (Invitrogen, USA) and fluorescence microscopy (Leica BMI4000, Germany). Liquid A (Calcein AM) and liquid B (EthD-1) of the kit were dissolved in PBS and mixed, then kept at room temperature for 40 min. Their working concentrations were 0.1 μl/ml and 2 μl/ml respectively. The printed tissue was washed twice with PBS and then mixed with a mixture of liquid A and liquid B. The mixture would submerge the printed tissue and be observed by fluorescence microscopy after 40 min at room temperature.

### Immunofluorescence analysis

Each group was fixed in 4% PFA for at least 30 min. Then the cells were collected by centrifuging at 1500 rpm for 5 min after cracked the composite hydrogels by using lysate (8.09 g sodium citrate, 4.39 g sodium chloride, 2.92 g EDTA, 500 ml deionized water) [17]. Washed the cells for 3 min 2 times with PBS and blocked for 30 min with 0.3% Triton X-100%. Then, the cells were washed with PBS once more. After permeabilization with 5% goat serum (Zsbio, China) at 37 °C for 30 min, the cells were incubated with primary antibodies overnight at 4 °C. After the cells were incubated with second antibodies for 2 h at room temperature, they were washed twice with PBS. Finally, the cells were incubated for 10 min with DAPI (1:300, Beyotime) as a nuclear stain. Images were scanned with fluorescence microscopy (Leica BMI4000, Germany) and a confocal microscope (Leica, TCSSP8, Germany). The antibodies used were as follows: keratin-8 (K8) (rabbit, 1:200, Abcam), K14 (mouse, 1:200, Abcam), K19 (rabbit, 1:200, Abcam), ATP1a1 (rabbit, 1:200, Abcam), estrogen receptor-α (ER-α) (rabbit, 1:200, Abcam), goat anti-rabbit Alexa Flour 488 (1:300, Beyotime), and goat anti-mouse Alexa Flour 488 (1:300, Beyotime), goat anti-rabbit Alexa Flour 594 (1:300, Beyotime), and goat anti-mouse Alexa Flour 594 (1:300, Beyotime).

### Quantitative real-time PCR

The cells collected by centrifuging at 1500 rpm for 5 min after cracked the composite hydrogels by using lysate were lysed in Trizol (Invitrogen). And the 200 μl chloroform per 1 ml Trizol were added and shaken for 15 s, then let the solution sit at room temperature for 3 min. Next, they were centrifuged at 12,000 rpm 4 °C for 15 min. The RNA contained in the aqueous phase was transferred to a new tube and was added 0.5 ml isopropanol per 1 ml Trizol. After being incubated for 10 min at room temperature, it was centrifuged at 12,000 rpm for 15 min at 4 °C. The RNA was centrifuged to the bottom of the tube. Total RNA was then reverse-transcribed with the PrimeScript^TM^RT reagent Kit (TaKaRa, China) and amplified with the TB Green^TM^ *Premix Ex Taq*^TM^ II (TaKaRa, China). The primers used were K8 (Fwd:ggacgaagcatacatgaacaagg, Rev: tgagatctgagactgcaactcac), K14 (Fwd: gtgagaaagtgaccatgcagaac, Rev: tgtagtctttgatctcagtgggc), ATP1a1 (Fwd:cgtgggtcttatctccatgattg, Rev:gtgattggatggtctcctgtaac), EDA (Fwd: ggacggcacctacttcatctata, Rev: caagtgttgtagttggtcttccc), NF-κb (Fwd: tgggactacacctctgcatatag, Rev: ggtcatagagaggctcaaagttc), and Shh (Fwd: ggagtctctacactatgagggtc, Rev: tgga ttcatagtagacccagtcg). The procedure of PCR with the Applied Biosystems 7500 Real-Time PCR System (Thermo Fisher Scientific) was an initiation for 30 s at 95 °C, followed by 40 thermal cycles each at 95 °C for 5 s and 60 °C for 34 s, and then dissociation analysis. All data were analyzed with the C(t) value comparison method.

### Statistical analysis

Each experiment was repeated independently at least three times. Data are expressed as means ± standard deviations. Differences between two groups or among multiple groups were analyzed by one-way ANOVA or two-way ANOVA. In one-way ANOVA, SNK-*q* tests were used in the comparison between each group. The statistical details were illustrated in each figure legends. A *p* value of < 0.05 was considered statistically significant.

## Results

### Identification of MPCs and screening differential expression markers between MPCs and SG cells

**Fig. 1.**
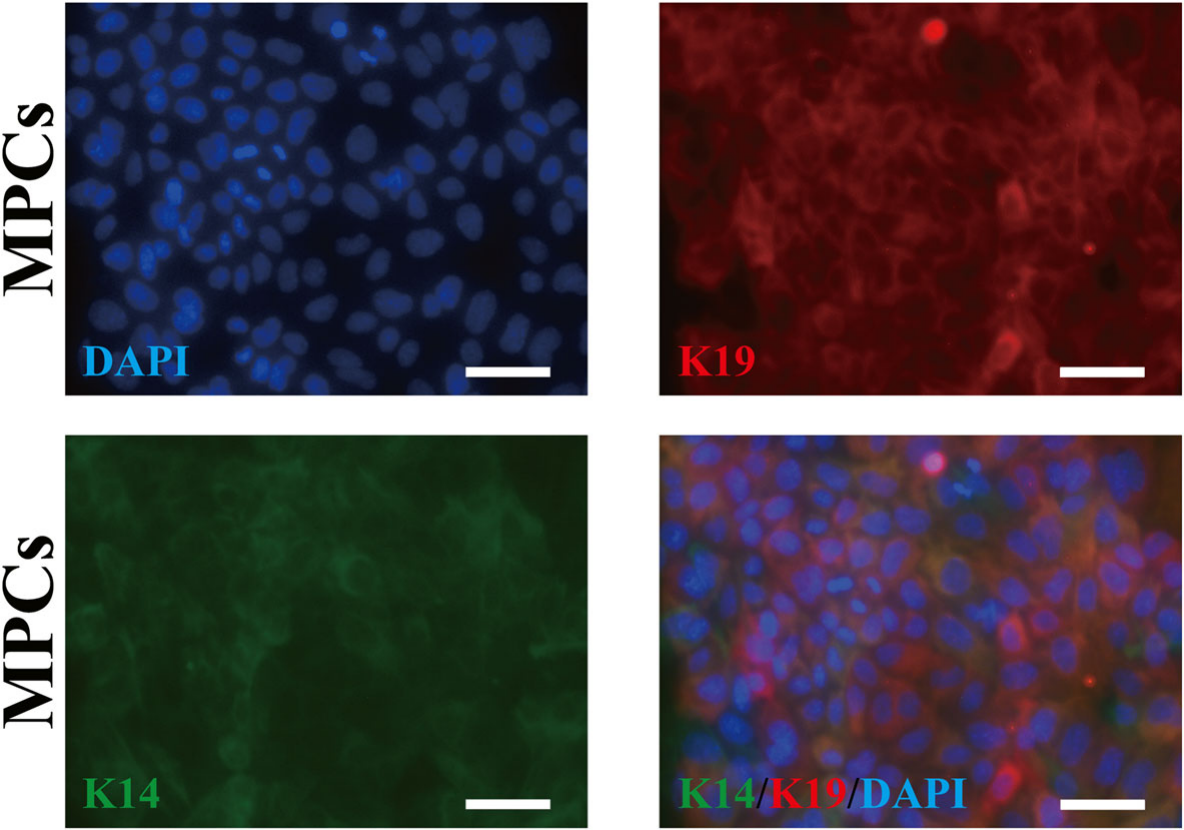
Identification of mammary progenitor cells (MPCs). Immunofluorescence staining of keratin-14 (K14) and keratin-19 (K19) of MPCs after isolated and cultured 1 day (scale bar, 50 μm)

After 3 days of cultures, the morphology assay under the microscope showed that the isolated primary MPCs could form the typical paving stone-like structure (Fig. 1). Immunofluorescence assay showed that mouse MPCs could express both K14 and K19, the epithelial-specific intermediate filament proteins [4], similar to human MPCs (Fig. 1). The expression of specific markers at the level of mRNA and protein could reflect the fate changes of progenitor cells [28]. To study whether the 3D bioprinted SG microenvironments could induce the differentiation of MPCs into SG cells, we have first screened the specifically expressed markers of SG cells which are not expressed in mammary gland cells. The sodium/potassium channel protein ATP1a1 has been demonstrated as the functional marker of mouse SG while not expressed in mammary glands [8]. Immunofluorescence assay showed consistent results, ATP1a1 displayed the greatest differential expression between SG cells and MPCs (Additional file 1: Figure S1).

### MPCs differentiate to SG cells in 3D bioprinted SG microenvironment

**Fig. 2.**
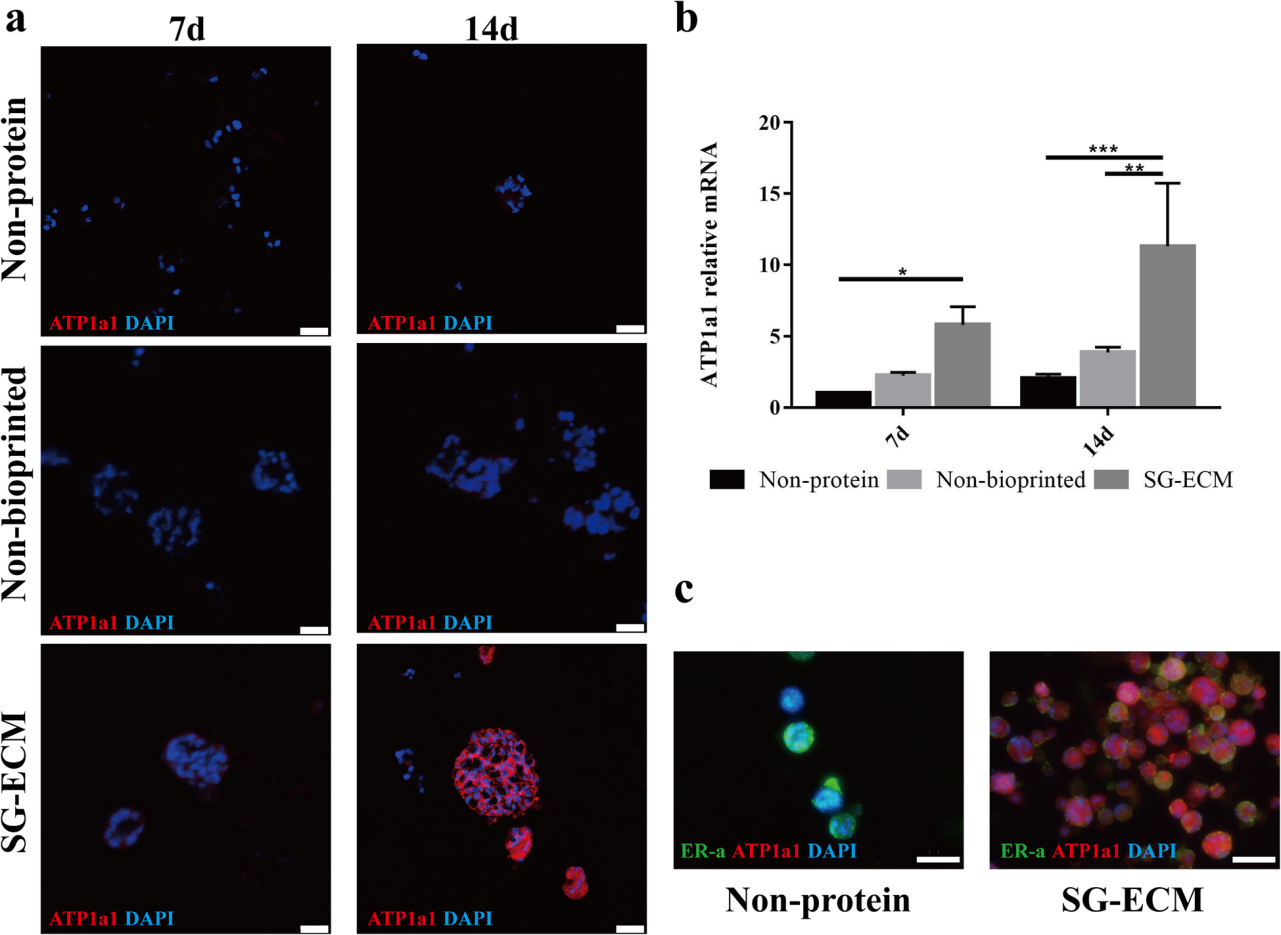
Mammary progenitor cells (MPCs) differentiate to sweat gland (SG) cells in three-dimensional (3D) bioprinted SG microenvironment. **a** Immunofluorescence staining of ATP1a1 of induced cells cultured in groups of SG extracellular matrix (SG-ECM), non-bioprinted and non-protein. Confocal images were taken at 7 days and 14 days after cultured (scale bar, 25 μm). **b** Gene expression of ATP1a1 of different groups. Data were presented as mean ± standard deviation (*n* = 3). In comparison, the two-way ANOVA analysis was used to detect the general difference between the time factors and grouping factors. The comparisons between each group were measured in each main factor’s one-way ANOVA analysis and further the SNK-*q* test. *p < 0.05, **p < 0.01. **c** Immunofluorescence staining of ATP1a1 and estrogen receptor-α (ER-α) of inducted cells after cultured 14 days (scale bar, 50 μm)

Using 3D bioprinting technology, we constructed an engineered SG microenvironment *in vitro*, which composed by mouse SG-ECM proteins and hydrogel material containing gelatin and sodium alginate and printed as a cylinder with a grid inside (Additional file 2: Figure S2a, b). The pore size of the 3D bioprinted structure is approximately 270 ± 22 μm, calculated through scanning electron microscopy (SEM) images (Additional file 2: Figure S2c), which conducive to the exchange of nutrients between cells and the environment [29, 30]. Viability/cytotoxicity assay further demonstrates that cells enclosed in the 3D bioprinted SG microenvironment can maintain high viability (Additional file 2: Figure S2d). Over time, compared with the control group, microphotographs showed that the 3D bioprinted SG microenvironment could promote cell proliferation and clusters formation even better (Additional file 2: Figure S2e). After being cultured for 7 and 14 days, the immunofluorescence and gene expression analysis revealed that the cells of SG-ECM group significantly expressed ATP1a1 compared with controls (Fig. 2a, b). In Fig. 2c, after being cultured for 14 days, the cells in SG microenvironment expressed ATP1a1 while having a low expression level of ER-α, which was the mammary gland specific marker. These data demonstrated that MPCs were successfully induced into SG cells by culturing in the 3D bioprinted SG microenvironment.

### MPCs mainly differentiate into luminal epithelial cells of SG in 3D bioprinted SG microenvironment

**Fig. 3.**
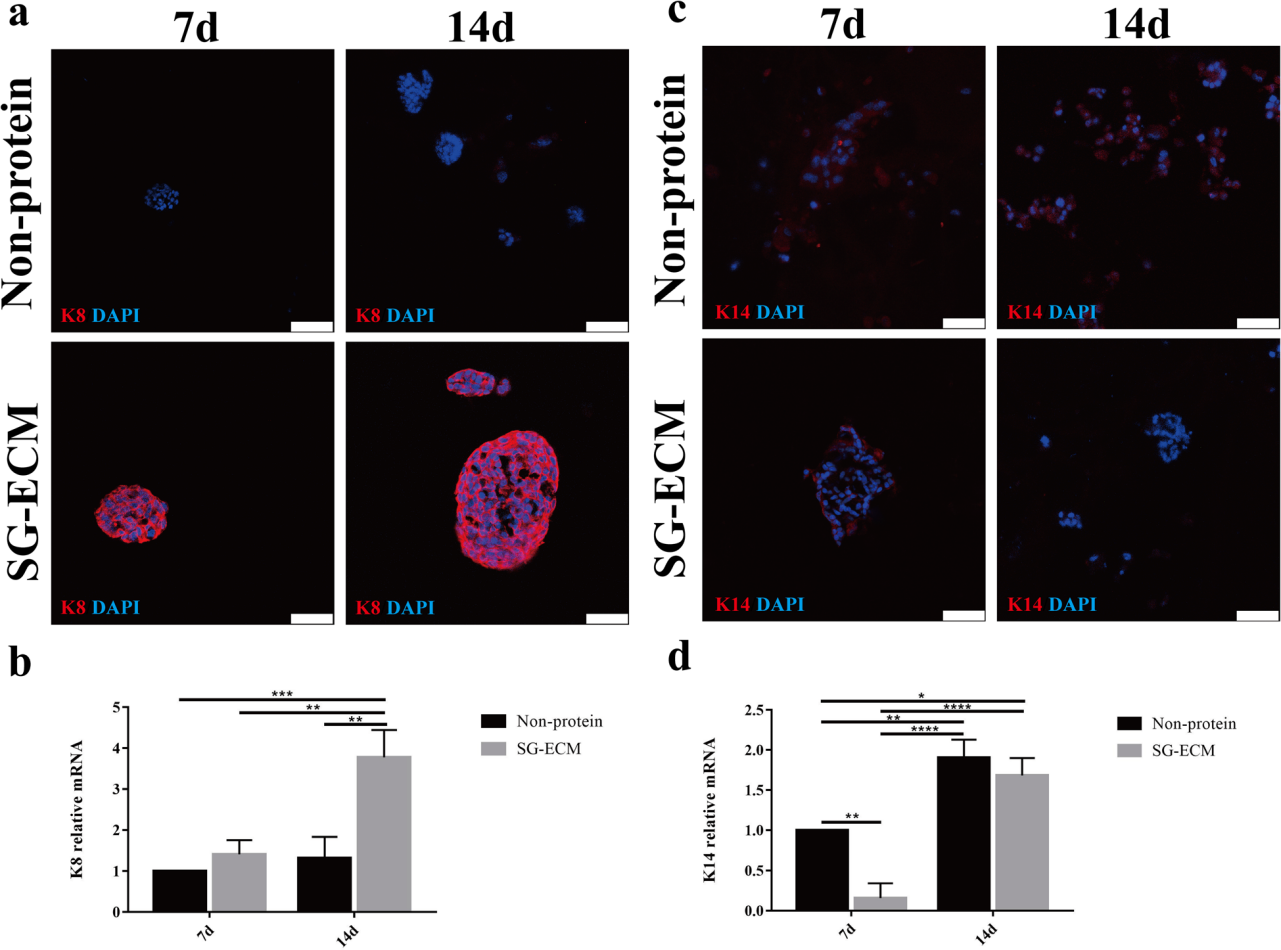
Differentiatied cells in three-dimensional (3D) bioprinted sweat gland (SG) microenvironment. **a**, **c** Immunofluorescence staining of keratin-8 (K8) and keratin-14 (K14) of inducted cells at 7 days and 14 days after bioprining (scale bar, 50 μm). **b**, **d** Gene expression of K8 and K14 in different groups. Data were presented as mean ± standard deviation (*n* = 3). The general difference was measured by the two-way ANOVA analysis. The comparisons between each group were analyzed by each main factor’s one-way ANOVA analysis and further the SNK-*q* test. *p < 0.05, **p < 0.01, ***p < 0.001

As mentioned above, MPCs express both K14 and K19. With the continuous development of the mammary gland, the luminal epithelial cells which are differentiated from MPCs still express K19, and the expression of K8 is gradually enhanced while the expression of K14 gradually weakened, which is opposite to the myoepithelial cells [4, 17]. In order to further study the differentiation direction of MPCs cultured in 3D bioprinted SG microenvironment, we detected the expression levels of K8 and K14 respectively. Compared with the control group, the cells of SG-ECM group had an increase in the expression of K8 after being cultured for 7 and 14 days (Fig. 3a, b). The expression of K14 was significantly decreased in the SG-ECM group, while increased in the controls (Fig. 3c, d).

**Fig. 4.**
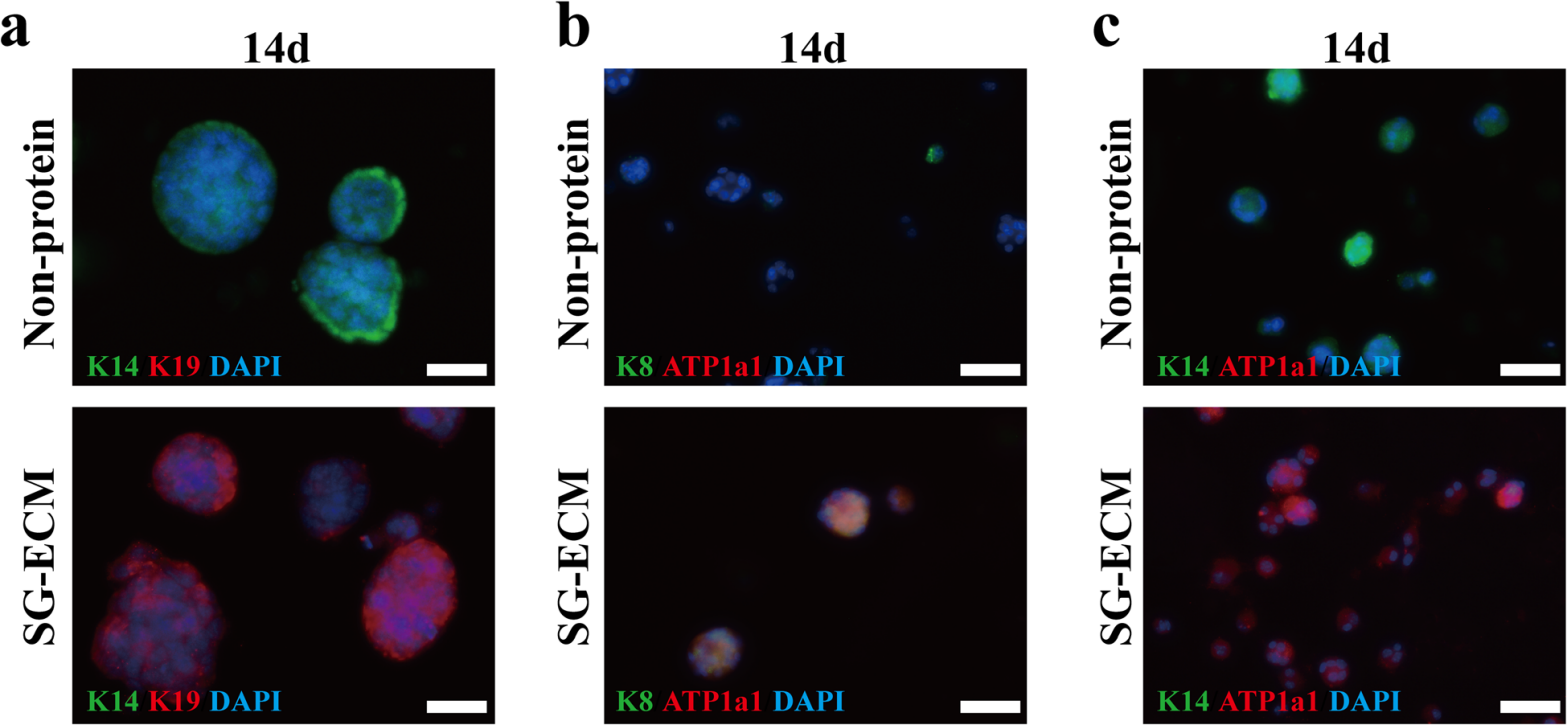
MPCs mainly differentiate into luminal epithelial cells of sweat gland (SG) in three-dimensional (3D) bioprinted SG microenvironment. **a** Immunofluorescence staining of keratin-14 (K14) and keratin-19 (K19) of inducted cells after cultured 14 days (scale bar, 50 μm). **b** Immunofluorescence staining of K8 and ATP1a1 of inducted cells after cultured 14 days (scale bar, 50 μm). **c** Immunofluorescence staining of K14 and ATP1a1 of inducted cells after cultured 14 days (scale bar, 50 μm)

We then examined the co-expression levels of K14 and K19, and the results showed that the cells which expressed K19 did not express K14 in SG-ECM group while in the controls it was just the opposite (Fig. 4a). The luminal epithelial cells of SG also express both K19 and K8 and do not express K14, which is similar to that of mammary gland cells [8, 23]. Immunoblot assays also revealed that both K8 and ATP1a1 were expressed in the cells of SG-ECM group while the controls expressed neither K8 nor ATP1a1 (Fig. 4b). The co-expression levels of K14 and ATP1a1 showed that ATP1a1 was only found in the SG-ECM group while controls only expressed K14 (Fig. 4c). Thus, this study suggested that MPCs tend to differentiate into luminal epithelial cells of SG by the direction of 3D bioprinted SG microenvironment.

### Shh signaling pathway involves in the differentiation of MPCs on 3D bioprinted SG microenvironment

**Fig. 5.**
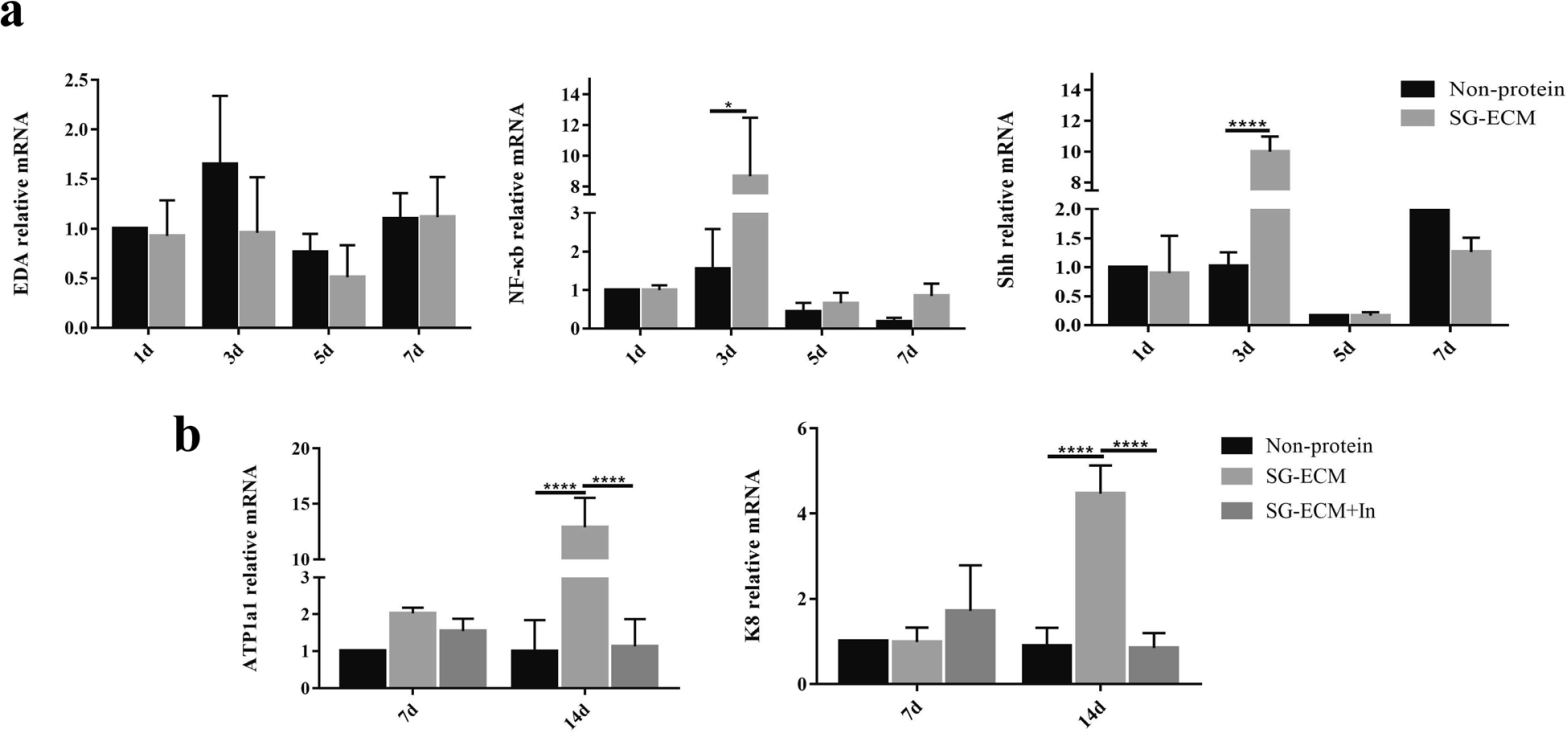
The Shh signaling pathway involves in the differentiation of mammary progenitor cells (MPCs) on three-dimensional (3D) bioprinted sweat gland (SG) microenvironment. **a** Gene expression of EDA, NF-κb, and Shh of different groups after cultured 1, 3, 5, and 7 days. **b** Gene expression of ATP1a1 and keratin-8 (K8) of different groups after cultured 7 and 14 days. Data were presented as mean ± standard deviation (SD) (*n* = 3). Data were presented as mean ± SD (*n* = 3). The general difference was measured by the two-way ANOVA analysis. The comparisons between each group were analyzed by each main factor’s one-way ANOVA analysis and further the SNK-*q* test. *p < 0.05, **p < 0.01, ***p < 0.001

In order to elucidate the mechanism by which MPCs tend to differentiate into luminal epithelial cells of SG in the 3D bioprinted SG microenvironment, we further tested the gene expression of EDA/NF-κb/Shh pathway, which participate in regulating the formation of secretory coil, at different time points and found that Shh signaling pathway had a significantly high expression at the third day during the induction progress (Fig. 5a). The expression of ATP1a1 and K8 was significantly suppressed when the inhibitor of the Shh signaling pathway was added on the third day of culture (Fig. 5b). These results revealed that the Shh signaling pathway was involved during the induction process of MPCs into SG in 3D bioprinted SG microenvironment.

## Discussion

Although previous reports have demonstrated the mammary niche redirected the differentiation of cells from other tissues, this study provides the first description, to our knowledge, of reprogramming MPCs using engineering microenvironment that was incorporating foreign tissues. Here, we show that MPCs can be induced and mainly differentiate into SG cells at both morphological and functional level. Quantitative RT-PCR combined with immunofluorescence analysis of keratin expression demonstrates that the 3D bioprinted SG microenvironment we created is more conducive to promoting the differentiation of MPCs into luminal epithelial cells of SG. Additionally, the Shh signaling pathway was involved in the induction process. These results strongly suggest the importance of the engineered microenvironment for redirecting differentiation of MPCs to regenerate foreign tissues.

Given the same origins of mammary gland cells and SG cells, they express plenty of similar keratins [8, 23]. Based on this, the functional protein ATP1a1 of SG cells was screened at the differential protein between the two cells, as well as ER-α which was specifically expressed in the mammary gland cells. Previous researches and our experimental data have shed light on that mouse MPCs expressed both K14 and K19, but we detected that induced MPCs in 3D bioprinted SG microenvironment expressed K8, K19, and ATP1a1, while we did not detect these cells colocalizing with K14 expression. These data prove that MPCs mainly differentiated into luminal epithelial cells of SG. Researchers have verified that the signaling pathway involved in SG development. It mainly includes Wnt, Eda, NF-κb, and Shh signaling pathway [23, 24]. To gain further insight into the mechanism of reprogramming, we examine which signaling pathways are highly expressed during induction of MPCs in 3D bioprinted SG microenvironment and find that Shh signaling pathway has a high expression at the third day of culture. Considering that the Shh signaling pathway does not exist in mature mammary glands but in SG, we use it as a screened pathway [25, 26]. As the result shows, the expression of ATP1a1 and K8 of cells cultured in 3D bioprinted SG microenvironment is significantly depressed after being handled with the inhibitor of Shh signaling pathway, which adds to the evidence that reprogramming of MPCs into SG cells is related to Shh signaling pathway.

The engineered SG microenvironment we create using gelatin-alginate hydrogels and the components from mouse SG-ECM proteins through 3D bioprinting approach in our study has been demonstrated in previous articles that it was capable of inducing epidermal progenitor cells into SG cells [19, 20]. It is capable of sustaining stem cells with long-term survival and differentiation because of the high cell compatibility and cell viability [19]. To fabricate a more tissue-specific artificial microenvironment for reprogramming MPCs into SG cells, the mouse SG-ECM proteins which are demonstrated that no residual SG cells in it are added into the tailored bioink (Additional file 2: Figure S2a). To date, engineering artificial microenvironment utilizing 3D bioprinting approach is being successfully used in many researches [15–17, 31, 32]. It can provide a 3D structure which has a high similarity to the natural microenvironment. To shed light on this question, we test that mouse SG-ECM proteins are unable to induce MPCs into SG cells in a 2D cultured environment (Additional file 3: Figure S3). Recent work has shed light on that the stiffness of ECM microenvironment can affect cell adhesion, migration, proliferation, and differentiation [33, 34]. ECM microenvironment with low stiffness promotes differentiation to the luminal epithelial cells by inhibiting the expression of RhoA signaling pathway, whereas MPCs more easily differentiate into myoepithelial cells in hard matrix [28, 35]. Therefore, we speculated that the stiffness of 3D bioprinted SG microenvironment we created in this study is more conducive to promoting the differentiation of MPCs into luminal epithelial cells.

## Conclusions

Taken together, the study provides clear evidence for the ability of differentiated mouse MPCs to regenerate SG cells by engineered SG microenvironment fabricated by gelatin-alginate hydrogels and mouse SG-ECM proteins through 3D bioprinting approach *in vitro*. Other studies have also shown that ECM with only chemical factors is not sufficient, and cell differentiation is also affected by many comprehensive factors, such as the structure and hardness of the matrix [36]. The significance of our results is proving the critical role of the engineered microenvironment in determining MPCs fate and cell function. Although its mechanism still needs to be further explored, the scheme we described is useful for regenerating damaged SG by MPCs and might provide a tool for inducing ideal cells or tissues *in vitro* through engineered microenvironment in the future.

## Additional files

Additional file 1: **Figure S1.** Screening of differential proteins between sweat gland (SG) and mammary progenitor cells (MPCs). Immunofluorescence staining of ATP1a1, ATP1b1, keratin-5 (K5) and keratin-19 (K19) of SG cells and MPCs in two-dimensional (2D) cultured environment (scale bar, 50 μm). (JPG 232 kb)

Additional file 2: **Figure S2.** Characteristics of three-dimensional (3D) bioprinted sweat gland (SG) microenvironment. (a) The content of DNA of centrifuged and Non-centrifuged mouse SG-extracellular matrix (ECM) proteins measured by spectrophotometer. The control was phosphate-buffered saline (PBS) which had no DNA (*n* = 3). The result demonstrated that there were no cells in the dermal homogenates. In the statistical analysis, one-way ANOVA was used to measure the difference between these three groups. In each group comparison, SNK-*q* test was used. *p < 0.05, **p < 0.01. (b) The process of 3D bioprinting with bioprinter. (c) The porous structure of 3D bioprinted SG microenvironment was observed using scanning electron microscopy (SEM) (scale bar, 100 μm). (d) Cell viability of the 3D bioprinted SG microenvironment. The live cells were labeled with Calcein AM and dead cells with EthD-1 (scale bar, 500 μm). (e) Cell morphology in groups of SG-ECM, Non-bioprinted and Non-protein at different time points (scale bar, 50 μm, 200 μm). (JPG 101 kb)

Additional file 3: **Figure S3.** Differentiation of mammary progenitor cells (MPCs) in two-dimensional (2D) cultured environment. (a) Immunofluorescence staining of ATP1a1 of induced cells cultured in 2D cultured environment without mouse sweat gland-extracellular matrix (SG-ECM) proteins. (scale bar, 50 μm). (b) Immunofluorescence staining of ATP1a1 of induced cells cultured in 2D cultured environment with mouse SG-ECM proteins. (scale bar, 50 μm). (c) Gene expression of ATP1a1 of different groups. The group of SG is positive control. Data were presented as mean ± standard deviation (*n* = 3). In the statistical analysis, one-way ANOVA was used to measure the difference between these three groups. In each group comparison, SNK**-***q* test was used. **p < 0.01. (JPG 47 kb)

## Data Availability

The datasets used and/or analyzed in the current study are available from the corresponding author upon reasonable request.

## Abbreviations

MPCs: Mammary progenitor cells
2D: Two-dimensional
3D: Three-dimensional
SG: Sweat gland
ECM: Extracellular matrix
PBS: Phosphate-buffered saline
PFA: Paraformaldehyde
SEM: Scanning electron microscopy
K14: Keratin-14
K19: Keratin-19
ER-α: Estrogen receptor-α
K8: Keratin-8
K5: Keratin-5
