# Supplementary figures and images for: Redirecting differentiation of mammary progenitor cells by 3D bioprinted sweat gland microenvironment
Source: Burns Trauma. 2019 Sep 23;7:29. doi: 10.1186/s41038-019-0167-y (PMC6755689; doi:10.1186/s41038-019-0167-y)

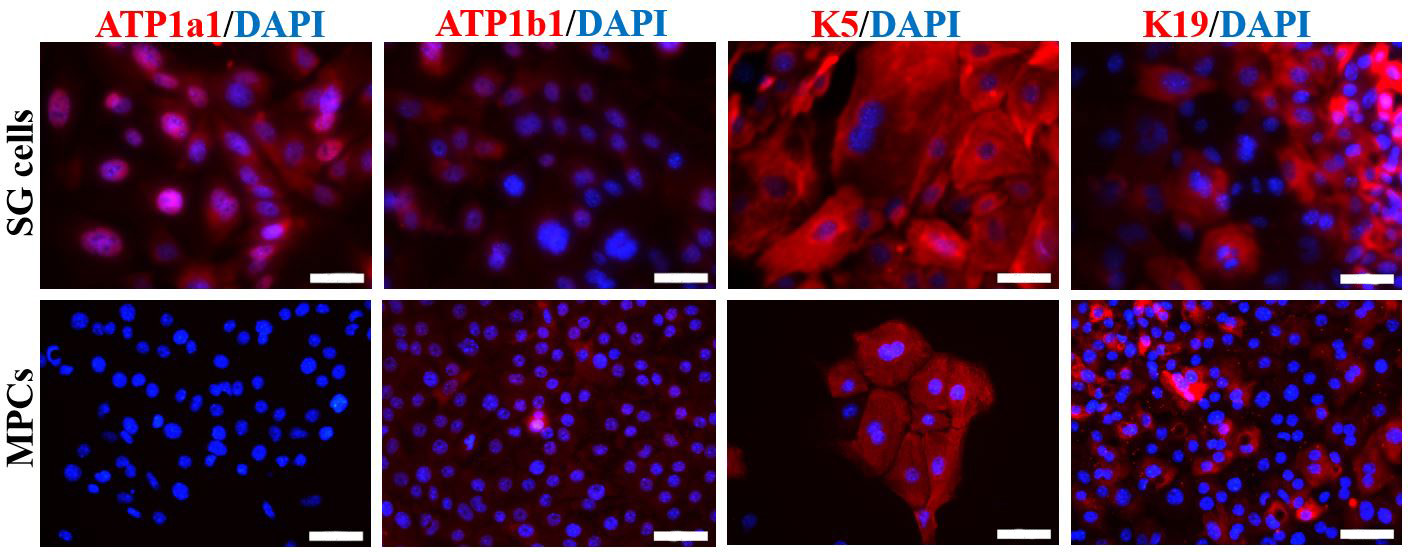

Supplement: Supplementary file 1 — Figure S1. Screening of differential proteins between sweat gland (SG) and mammary progenitor cells (MPCs). Immunofluorescence staining of ATP1a1, ATP1b1, keratin-5 (K5) and keratin-19 (K19) of SG cells and MPCs in two-dimensional (2D) cultured environment (scale bar, 50 μm). (JPG 232 kb) [file 41038_2019_167_MOESM1_ESM.jpg]

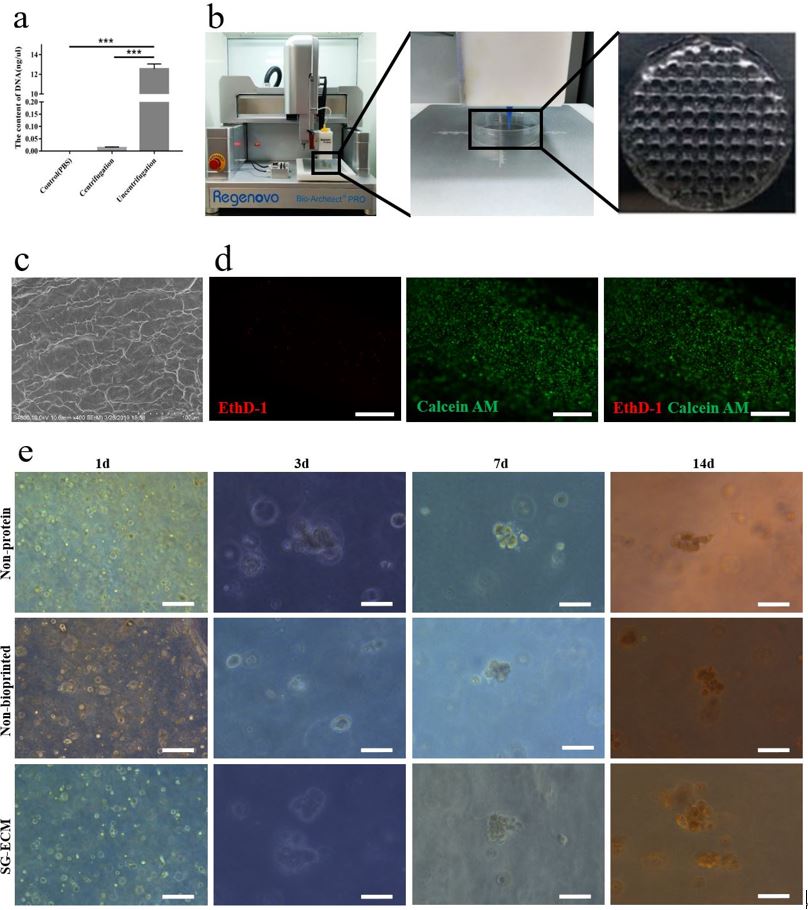

Supplement: Supplementary file 2 — Figure S2. Characteristics of three-dimensional (3D) bioprinted sweat gland (SG) microenvironment. (a) The content of DNA of centrifuged and Non-centrifuged mouse SG-extracellular matrix (ECM) proteins measured by spectrophotometer. The control was phosphate-buffered saline (PBS) which had no DNA (n = 3). The result demonstrated that there were no cells in the dermal homogenates. In the statistical analysis, one-way ANOVA was used to measure the difference between these three groups. In each group comparison, SNK-q test was used. *p < 0.05, **p < 0.01. (b) The process of 3D bioprinting with bioprinter. (c) The porous structure of 3D bioprinted SG microenvironment was observed using scanning electron microscopy (SEM) (scale bar, 100 μm). (d) Cell viability of the 3D bioprinted SG microenvironment. The live cells were labeled with Calcein AM and dead cells with EthD-1 (scale bar, 500 μm). (e) Cell morphology in groups of SG-ECM, Non-bioprinted and Non-protein at different time points (scale bar, 50 μm, 200 μm). (JPG 101 kb) [file 41038_2019_167_MOESM2_ESM.jpg]

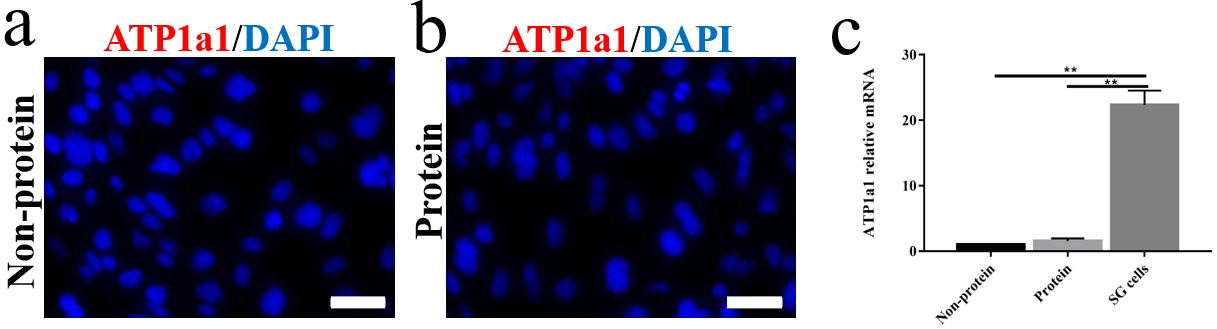

Supplement: Supplementary file 3 — Figure S3. Differentiation of mammary progenitor cells (MPCs) in two-dimensional (2D) cultured environment. (a) Immunofluorescence staining of ATP1a1 of induced cells cultured in 2D cultured environment without mouse sweat gland-extracellular matrix (SG-ECM) proteins. (scale bar, 50 μm). (b) Immunofluorescence staining of ATP1a1 of induced cells cultured in 2D cultured environment with mouse SG-ECM proteins. (scale bar, 50 μm). (c) Gene expression of ATP1a1 of different groups. The group of SG is positive control. Data were presented as mean ± standard deviation (n = 3). In the statistical analysis, one-way ANOVA was used to measure the difference between these three groups. In each group comparison, SNK-q test was used. **p < 0.01. (JPG 47 kb) [file 41038_2019_167_MOESM3_ESM.jpg]
